# Density Mediates the Predator-Induced Growth and Metamorphic Plasticity of Chinhai Spiny Newt Larvae

**DOI:** 10.3390/ani14101510

**Published:** 2024-05-20

**Authors:** Xihong Zhu, Xia Qiu, Wei Li, Shiyan Feng, Aichun Xu

**Affiliations:** 1 College of Life Sciences, China Jiliang University, Hangzhou 310018, China; zxh0531@cjlu.edu.cn (X.Z.); lw0512@cjlu.edu.cn (W.L.); fsy0714@cjlu.edu.cn (S.F.); 2 Institute of Endangered Animals and Biodiversity, China Jiliang University, Hangzhou 310018, China

**Keywords:** *Echinotriton chinhaiensis*, conspecific density, predator-induced plasticity, endangered species

## Abstract

**Simple Summary:**

In this study, we investigated how predators and conspecific density impact the growth of Chinhai spiny newt larvae. Using a two-factor experimental design, we manipulated both factors to explore their independent and interactive effects on larval development. Our findings demonstrate that both high and low conspecific densities constrain larval growth, while predators also limit growth. Interestingly, high conspecific density restricts predator-induced growth plasticity without interacting effects. This study provides valuable insights into the complex interplay between environmental factors and larval development in endangered Chinhai spiny newts. Understanding these dynamics is crucial for informing conservation strategies aimed at protecting this vulnerable species.

**Abstract:**

Predators significantly influence amphibian larval development. Predator-induced plasticity is often studied independently from conspecific density effects, but these environmental factors may interact. We conducted two-factor factorial experimental design to manipulate conspecific density and predator cues, aiming to investigate the independently or interactive impacts of these two factors on the development of Chinhai spiny newt larvae (*Echinotriton chinhaiensis*). Our findings reveal that both high and low conspecific densities constrain spiny newt larval growth and predators also limit growth. Interestingly, high conspecific density restricts predator-induced growth plasticity without interacting effects. Only lower density groups exhibit slower growth responses to predators. Our study investigates how density mediates predator-induced plasticity in the endangered Chinhai spiny newt larvae, providing insights into their intricate life history. These results contribute to the understanding of predator-induced plasticity in amphibians and provide insights into the adaptive strategies of endangered species like Chinhai spiny newt. Such knowledge informs the development of effective conservation strategies for their protection.

## 1. Introduction

Predator-induced plasticity is a crucial aspect of understanding the complex life history of amphibians. Predators have a significant impact on the development process in larval amphibians, leading to two main metamorphic outcomes. Firstly, many studies have demonstrated that the majority of larvae may experience the same or delayed time to metamorphosis in response to the presence of predators [1,2]. For instance, tadpoles of the red-legged frog, *Rana aurora*, prolong their larval period and increase in size at metamorphosis when predators are present [3]. Secondly, a few studies suggest that larvae may undergo an earlier time to metamorphosis in response to the presence of predators [1,2]. For example, tadpoles of *Hyla intermedia* [4] exhibit a reduced time to metamorphosis and may end up smaller or the same size at metamorphosis.

Density can indeed influence predator-induced plasticity. The dilution effect theory suggests that, as group size increases, the likelihood of individual predation decreases, leading individuals to invest less in antipredator responses [5,6]. For example, tadpoles of the toad (*Bufo maculatus*) form colonies to reduce predator pressure [7]. However, higher density also escalates competition for resources such as food and habitat, significantly impacting the growth and development of amphibian larvae. For instance, high density can prolong metamorphosis and result in smaller body sizes in *Litoria aurea* tadpoles [8]. In the growth and development of prey, predators and intraspecific density typically involve a trade-off, with density potentially reducing predator-induced plasticity [9,10,11]. For example, predators induce larger tails and shorter guts of larval wood frogs, *R. sylvatica*, while high density decreases these responses [12]. Understanding the combined effects of these two factors is crucial for unraveling the predator-induced plasticity and metamorphic decisions employed by amphibians. Moreover, such studies may offer valuable biological insights for the implementation of in situ conservation and preservation efforts, particularly for endangered species.

The Chinhai spiny newt, *Echinotriton chinhaiensis*, is one of the critically endangered salamander species, with limited numbers found in the eastern part of Zhejiang province, China. Since 2004, it has been classified as “Critically endangered” on the IUCN Red List of Threatened Species due to the rarity of natural populations [13]. Predator risk and intraspecific competition during early development emerge as key factors contributing to the endangered status of the Chinhai spiny newt [14]. The suitable habitat for larval newts is highly specialized and limited, consisting of still-water ponds situated at the border of agricultural and forested areas, typically with sizes of approximately two square meters. Based on field observations, these ponds host a notable population of dragonfly nymphs, estimated at approximately five individuals per square meter. Due to the limited size of the ponds, these dragonfly nymphs pose a significant predation threat to the Chinhai spiny newt larvae. Furthermore, the density of Chinhai spiny newt larvae in the ponds is exceptionally high, reaching a maximum of 1800 individuals per square meter according to field observations. This high density leads to intense competition for resources, significantly impacting their survival. Therefore, investigating how the Chinhai spiny newt adjusts its response to predator risk based on density during early development stages will enhance our comprehension of the species’ early life history. Given its endangered status, this understanding is crucial for the effective protection of the Chinhai spiny newt.

Here, we conducted two-factor factorial experimental design to manipulate conspecific density and predator cues, aiming to investigate the impact of these two factors on the development of Chinhai spiny newt larvae. Our objectives are (1) to determine whether density affects the growth and metamorphic traits of Chinhai spiny newt larvae and (2) to ascertain whether high density reduces the predator-induced growth or metamorphic response in larvae. We predict that (1) both density and predators will separately impact the growth and metamorphic traits of spiny newt larvae and (2) the predator-induced growth or metamorphic response will decrease as density increases.

## 2. Materials and Methods

### 2.1. Animal Collection and Maintenance

In early May 2023, we collected 20 clutches of freshly laid eggs of the Chinhai spiny newt from the Ruiyansi Forest Park (29°48′24″ N, 121°51′12″ E) in the Ningbo municipality, China. Eggs were transported to our laboratory located within the park, where these eggs were maintained in their respective clutches until hatching. The hatching process was completed under natural photoperiod and temperature conditions in a cylindrical plastic tank (7.5 cm high and 20 cm in diameter). In each hatching tank, 1.5 cm of water was added and a regular water-absorbing sponge (17 × 12 × 2 cm) was placed in the water, ensuring that it remained in full contact with the water to maintain the humidity required for egg hatching. The larvae were reared in 20 rectangular plastic tanks (45 × 29.5 × 14.5 cm) with 4.5 cm of water. All water used in the experiment was collected from the ponds where the eggs were collected.

Dragonfly larvae (*Polycanthagyna ornithocephala*), a native predator of the Chinhai spiny newt larvae, were collected from the ponds near the egg collection location. To avoid size-related effects, all selected dragonfly larvae were individuals of similar size (length: mean ± SD = 4.53 ± 0.13 cm, mass: mean ± SD = 1.53 ± 0.07 g) in the late-instar stage. Dragonfly larvae were maintained in a plastic tank (85 × 56 × 18 cm) with aquatic plants and water from the collection location. Aquatic plants were placed to provide refugia for the dragonfly larvae. We fed tubifex worms to predators once a day before the experiment.

### 2.2. Experiment Design

We simulated different density treatments and predator treatments to evaluate the responses of the Chinhai spiny newt larvae to density, predator risk, and the interaction between the two. A net cage (14.5 × 16 × 15 cm, mesh size: 1 mm) containing a dragonfly larva was placed inside the rearing tank of the newt larvae to simulate a predator condition. The net cage allowed predator cues to be transmitted but did not allow true predation. Since Chinhai spiny newt is an endangered species, we cannot use the larvae to feed dragonfly larvae; we collected another prey of the dragonfly larva, tadpoles of the Zhenhai wood frog (*R. zhenhaiensis*), which coexist with newt larvae in the same ponds. These tadpoles were used as food during experiments for the dragonfly larvae to stimulate the release of predator cues by the predators. At the same time, the wood frog tadpoles likely released alarm cues, which might also alert the spiny newt larvae. We acknowledge that this may reduce the effect but, in some empirical studies, this has been shown to be effective [15,16,17].

The medium-density group was set at 15 individuals per box (with a box water volume of approximately 0.006 cubic meters), based on the mean actual field density (2571 individuals per cubic meter). Experimental groups below the average density were set as the low-density group at 5 individuals per box, while those above the average density were set as the high-density group at 30 individuals per box. Each density group was randomly assigned to either the predator treatment group (present) or the control group (absent), creating combined experimental groups that investigated the interaction between density and predator presence. Each experimental group was repeated four times, forming a total of 24 experimental units (three densities groups × two predator treatments × four replicates). We arranged the treatments using a randomized block design, with each of the four blocks containing one tank from each treatment combination. By comparing the differences in the growth and metamorphic traits of spiny newt larvae in different density groups under the same predator conditions, we can determine the impact of density on spiny newt larvae; by comparing the growth and metamorphic traits of spiny newt larvae in different predator conditions under the same density groups, we can determine the impact of predator risk on spiny newt larvae. In addition, by comparing the growth and metamorphic traits in each interactive experimental group, we can explore whether the impact of predator risk will decrease as population density increases.

### 2.3. Experiment Procedure

**Figure 1:**
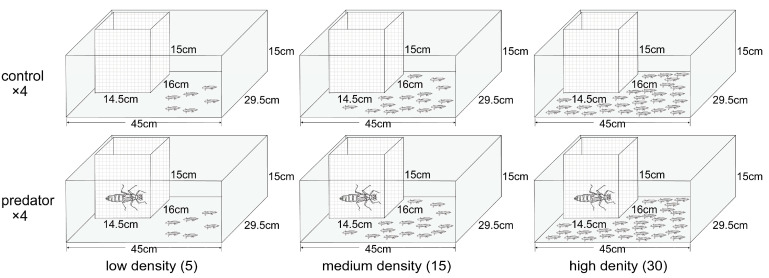
Schematic diagram illustrating the experimental design.

We randomly transferred one, three, or six newt larvae hatched on the same day from each clutch into each tank, resulting in 5, 15, or 30 larvae per tank. After eight rearing tanks of identical density were set up, with an empty net cage placed in one corner of each tank, a 10-day acclimation period was initiated. During this acclimation period, the larvae were fed brine shrimp (2 mL) three times a day. The predator treatments were initiated after the 10-day acclimation. Four tanks of each density were randomly assigned to either the predator-present or predator-absent treatment. In the predator-present treatment group, a dragonfly larva was placed in the net cages, while the net cages remained empty in the predator-absent treatment group (Figure 1). A total of 12 dragonfly larvae were randomly placed in each predator-present tank. Dragonfly larvae were fed wood frog tadpoles once a day during this period to ensure they released sufficient predator cues. Spiny newt larvae were fed brine shrimp (3 mL) three times a day to ensure sufficient food. The rearing water was replaced every day, and the temperature ranged from 21 °C to 23 °C during the rearing period.

Measurements of growth and metamorphic traits were taken for each individual. For growth, we measured length and mass on the first of the experiments, as well as on the 21st day, just before the larvae came to metamorphosis. The growth rate is defined as the increase in length and mass over these 21 days. Length was defined as the distance from the snout to the tip of the tail in a spiny newt larva. Each individual was photographed using a digital camera (Company Zhongweikechuang, Shenzhen, China, type ZW-U500) controlled by S-EYE software (version 1.4.4.500), with a 0.5 cm scale bar included in the photos for length calibration. Length was taken from the photos using Image J software (version 1.53 e) to the nearest 0.001 cm. Mass was measured to the nearest 0.0001 g using an electronic balance (Company G&G, Changshu, China, type G&G JJ224BF) after blotting the larvae quickly with absorbent paper. Upon completing the measurements, all individuals were returned to their original rearing tank.

For metamorphic traits, we measured length and mass at metamorphosis, time to metamorphosis, and survival to metamorphosis. We measured the length and mass of each individual at the day of metamorphosis using the same measurement methods as those used for growth measurements. Time to metamorphosis is defined as the duration from hatching to emergence onto land and the loss of external gills in Chinhai spiny newt larvae. The survival to metamorphosis is defined as the proportion of Chinhai spiny newt larvae that successfully reached metamorphosis. Any individuals that did not metamorphose all died during the process of reaching metamorphosis.

### 2.4. Statistical Analysis

We used linear mixed models (LMM) and generalized mixed models (GLMM) from the R package *lme4* [18], along with analysis of variance (ANOVA), to examine variations in growth and metamorphosis among Chinhai spiny newt larvae across different density treatments and predator treatments. As we were unable to track the initial length (or mass) of each individual as well as the length (or mass) on the 21st day, we first compared the length and mass between treatment groups at the beginning of the experiment. We did not find significant differences in neither length nor mass among treatment groups at the start of the experiment (see Supplementary Tables S1 and S2). Based on these results, we defined the growth rate as the increase in length or mass within 21 days, calculated as the length (or mass) on the 21st day minus the average initial length (or mass), divided by 21 days. Furthermore, we directly used the measurements of length (and mass) taken on the 21st day as an additional indicator of growth rate to validate the accuracy of our calculated growth rates. We considered growth rate as the dependent variable and density treatments, predator treatments, and the interaction of density and predator treatments as fixed effects, while assuming a Gaussian error distribution. To account for the variance of within-treatment, we treated replicates as a random effect. The significance of models was assessed with the “*anova*” function applying type III sums of squares calculations to test interaction terms. If a significant interaction exists, we will compare the variations between different densities within predator treatments separately and compare the variations between predator treatments within density treatments. Pairwise comparisons were performed using the “*emmeans*” function from *emmeans* package to examine differences within predator treatment and density treatment groups.

For metamorphic traits, we performed the same analysis for length and mass measured at metamorphosis; we considered length (or mass) as the dependent variable and density treatments, predator treatments, and the interaction of predator and density treatments as fixed effects, while assuming a Gaussian error distribution. To account for the variance of within-treatment, we treated replicates as a random effect, and the process of interaction terms test and pairwise comparison is similar to the previous one. For time to metamorphosis, we performed a similar analysis but assumed a Poisson error distribution. We also performed a similar analysis for survival to metamorphosis, assuming a Binomial distribution, treating survival as a binary dependent variable, with “1” representing survival and “0” representing died.

## 3. Results

### 3.1. Growth Rate

**Figure 2:**
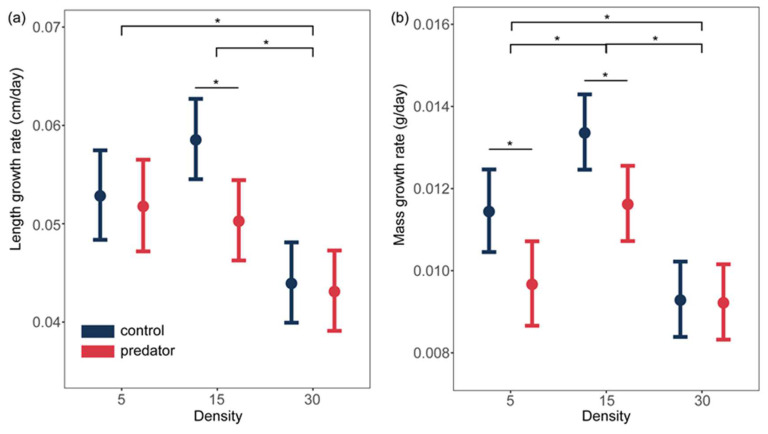
Effects of density treatments and predator treatments on the (**a**) length growth rate and (**b**) mass growth rate of the larval Chinhai spiny newts are depicted. Colorful points are the predicted average value and error bars represent the 95% confidence interval. “*” indicates significant difference.

Results of ANOVA showed that both density and predator presence significantly affect the growth rate of spiny newt larvae (Table 1, Table 2, Table 3, Tables S4 and S5, and Figure 2). However, there were no interaction effects between density treatments and predator treatments on the larvae growth (Table 1 and Table S3). Firstly, the larvae in the high-density group exhibited significantly slower growth in both length (Table 4, estimate = −0.009, t = −2.870, *p* = 0.014; estimate = −0.015, t = −4.983, *p* < 0.001) and mass (Table 4, estimate = −0.002, t = −3.125, *p* = 0.008; estimate = −0.004, t = −6.194, *p* < 0.001) compared to the lower density groups. And, compared to the medium-density group, the growth rate of larvae in the low-density group was significantly slowed down in mass (Table 4, estimate = 0.002, t = 2.777, *p* = 0.012). Secondly, in the presence of predators, larvae in the low-density and medium-density groups exhibited slower growth in both length (Table 4, estimate = −0.008, t = −2.823, *p =* 0.012) and mass (estimate = −0.002, t = −2.429, *p =* 0.023, estimate = −0.002, t = −2.644, *p =* 0.018). However, this inducible growth response did not occur in larvae from the high-density group (Table 3 and Figure 2). In addition, we found similar results with the additional growth indicators with length and mass on the 21st day (Tables S3–S5).

### 3.2. Metamorphic Traits

**Figure 3:**
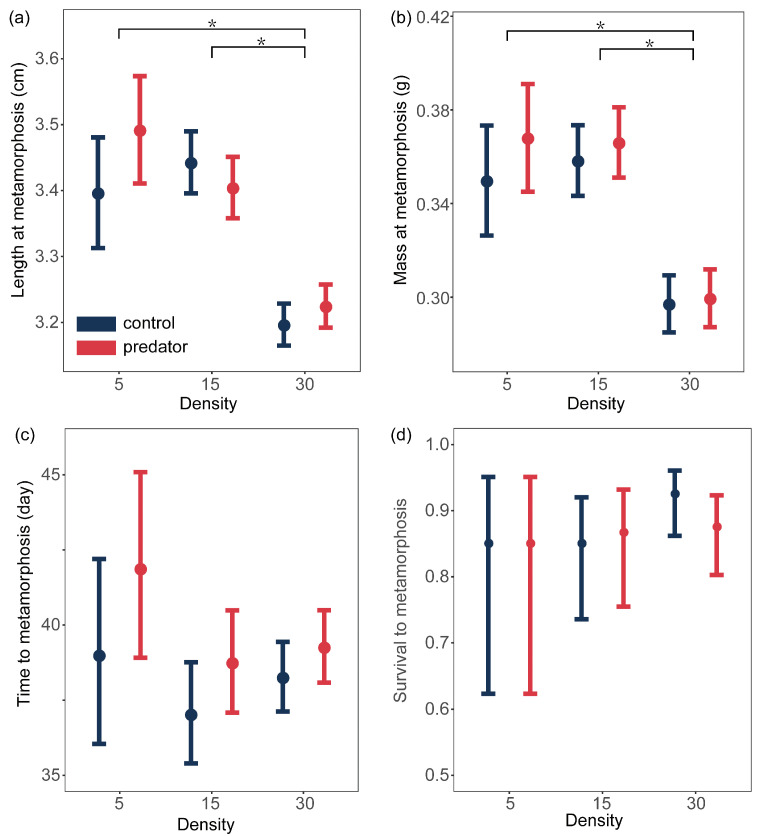
Effects of density treatments and predator treatments on metamorphic traits of the larval Chinhai spiny newts are depicted: (**a**) length at metamorphosis; (**b**) mass at metamorphosis; (**c**) time to metamorphosis; (**d**) survival to metamorphosis. Colorful points are the predicted average value and error bars represent the 95% confidence interval. “*” indicates significant difference.

Results of ANOVA showed that both density and predator presence significantly affect the metamorphic traits of spiny newt larvae (Table 1, Table 2 and Table 3 and Figure 3). However, there were no interaction effects between density treatments and predator treatments on the larvae metamorphosis (Table 1 and Figure 3). Firstly, the larvae in the high-density group exhibited significantly smaller size at metamorphosis in both length (Table 4, estimate = −0.200, t = −4.381, *p* < 0.001; estimate = −0.246, t = −8.521, *p* < 0.001) and mass (Table 4, estimate = −0.053, t = −3.91, *p* < 0.001; estimate = −0.061, t = −6.199, *p* < 0.001) compared to the lower density groups. We did not find a significant difference in the body size at metamorphosis of larvae between the low-density group and the medium-density group (Table 2). We also did not find any differences in the time and survival to metamorphosis of spiny newt larvae among the density treatments (Table 2). Secondly, although ANOVA results showed a significant difference in the time to metamorphosis of larvae among the predator treatments (Table 1), the pairwise comparisons did not detect any differences (Table 3). Because pairwise comparisons conducted with “*emmeans*” may not always reach statistical significance. This discrepancy could stem from the correction methods used in pairwise comparisons (e.g., Bonferroni correction), which increase stringency and reduce the likelihood of detecting differences. We also did not find any significant differences in survival to metamorphosis either among density treatments or among predator treatments (Table 2 and Table 3).

## 4. Discussion

Our research sheds light on how density mediates predator-induced plasticity in the endangered Chinhai spiny newt larvae. High conspecific density constrained the growth rate and body size at metamorphosis of spiny newt larvae but did not impact their time or survival to metamorphosis. Low conspecific density also constrained the growth rate of spiny newt larvae, without affecting their metamorphic traits. The presence of predators also constrained the growth rate of spiny newt larvae, without affecting their metamorphic traits. Interestingly, our results showed that larvae exhibited slower growth responses to the presence of predators only in lower density groups, not in the high-density group, indicating that high conspecific density constrained predator-induced growth plasticity. Furthermore, we did not find any interaction effects between density and predators on the growth or metamorphosis of Chinhai spiny newt larvae.

We demonstrate that the conspecific density constrained the growth of spiny newt larvae. Firstly, compared to the two lower density groups, the growth rate of larvae in the high-density group was significantly slowed down (Figure 2), as well as exhibiting a smaller size at metamorphosis (Figure 3). This demonstrates that the high conspecific density constrained both the growth rate and metamorphic size of spiny newt larvae. Reduced growth rate is a common response to increase in density in other amphibian larvae, such as *Argenteohyla siemersi pederseni* tadpoles [19]. As density increases, the resources available to the individual are reduced and there is not enough energy for growth and development, which mediate growth and developmental restriction, as well as affect its traits at metamorphosis [20]. Secondly, compared to the medium-density group, the growth rate of larvae in the low-density group was significantly slowed down (Figure 2), without affecting metamorphic traits (Figure 3). This demonstrates that the low conspecific density also constrained the growth rate of spiny newt larvae. In this experiment, there was an adequate supply of food. One possible explanation for this phenomenon is due to Allee effects, which believe that low conspecific density can negatively impact individual development. At lower densities, food may not be as well stirred and suspended as at higher densities, leading to reduced accessibility and, subsequently, hindering larval growth [21]. For example, the tadpoles of *Rhacophorus moltrechti* in the lower density group exhibited a lighter mass at metamorphosis [22].

The presence of predators limited the growth rate of Chinhai spiny newt larvae. In the two lower density groups, the growth rate of spiny newt larvae under predator-present conditions was significantly slowed down compared to the larvae in predator-absent conditions, indicating that the presence of predators affected larval growth. Many studies have found that the presence of predators induces a decrease in the growth rate of amphibian larvae, delaying or maintaining the same time to metamorphosis [23,24], for instance, *Ambystoma opacum* and *Triturus alpestris* [25,26]. This is similar to our results. One possible explanation is that the presence of predators reduces the activity levels (e.g., foraging behavior) of prey animals and affects foraging, resulting in constraints in growth rate [27,28,29]. It is also possible that predator cues can increase metabolic and physiological costs, which mediate the growth and developmental restriction [30,31]. For instance, in *R. lessona*, physiological mechanisms such as intestinal emptying influence growth rate [31].

Interestingly, we demonstrate that high conspecific density constrained predator-induced growth plasticity of Chinhai spiny newt larvae without interacting effects, which aligns with our prediction. Our results reveal that spiny newt larvae exhibited slower growth responses to the presence of predators only in two lower density groups, not in the high-density group (Figure 2). This result demonstrates that high density reduces the predator-induced growth plasticity of Chinhai spiny newt larvae. This is consistent with “dilution effect”, which suggests that prey animals can reduce the predator risk borne by each individual by aggregating to form groups [10]. Many studies have yielded similar results. For instance, tadpoles of leopard frog, *R. pipiens*, did not respond to predators when exposed to high density [32]. In addition, the total attack rate of predators when *Phrynomantis microps* are aggregated is significantly lower than when they are randomly distributed, which also proves the “dilution effect” [33]. Furthermore, we did not find any interaction effects between density and predators on the growth or metamorphosis of Chinhai spiny newt larvae. This has been verified in multiple studies [34,35]. For example, density and predator presence did not have interacting effects on the metamorphic traits (include survival, mass, and time at metamorphosis) [36].

## 5. Conclusions

Our study reveals that density mediates predator-induced plasticity in the endangered Chinhai spiny newt larvae. Both high and low conspecific densities constrained the growth of spiny newt larvae, while the presence of predators also had a constraining effect on their growth, without affecting their metamorphosis. Interestingly, our results demonstrate that high conspecific density constrained predator-induced growth plasticity, without interacting effects. However, using net cages to simulate predator cues in experimental design may have limitations, as it cannot fully replicate the natural interactions between predators and prey in their native habitat. This enriches the theory of predator-induced plasticity in amphibians and contributes to understanding the adaptive strategies of endangered amphibians such as the Chinhai spiny newt in response to environmental changes. This research provides valuable insights for developing effective conservation strategies for the Chinhai spiny newt.

## Figures and Tables

**Table 1:** The effects of density treatments, predator treatments, and their interaction on growth rate and metamorphic traits of Chinhai spiny newt larvae, shown as type III analysis-of-deviance tables. Significant differences (*p* < 0.05) between different groups are highlighted in bold.

Response	Fixed Effect	X^2^	*df*	*P*
Length growth rate	**Density treatments**	**30.453**	**2**	**<0.001**
	**Predator treatments**	**4.125**	**1**	**0.042**
	Density treatments × Predator treatments	4.027	2	0.134
Mass growth rate	**Density treatments**	**48.946**	**2**	**<0.001**
	**Predator treatments**	**8.661**	**1**	**0.003**
	Density treatments × Predator treatments	4.252	2	0.119
Length at metamorphosis	**Density treatments**	**136.897**	**2**	**<0.001**
	Predator treatments	0.678	1	0.410
	Density treatments × Predator treatments	4.649	2	0.098
Mass at metamorphosis	**Density treatments**	**98.809**	**2**	**<0.001**
	Predator treatments	1.052	1	0.305
	Density treatments × Predator treatments	0.726	2	0.696
Time to metamorphosis	Density treatments	4.411	2	0.110
	**Predator treatments**	**4.378**	**1**	**0.036**
	Density treatments × Predator treatments	0.708	2	0.702
Survival to metamorphosis	Density treatments	1.742	2	0.419
	Predator treatments	0.569	1	0.451
	Density treatments × Predator treatments	1.134	2	0.567

**Table 2:** Pairwise comparisons were conducted to evaluate the impact of density treatments on growth rate and metamorphic traits of Chinhai spiny newt larvae within each predator treatment group. Significant differences (*p* < 0.05) between different groups are highlighted in bold.

Predator Treatments	Contrast	Estimate	SE	t/z Value	*p* Value
Length growth rate					
Averaged	5 vs. 15	0.002	0.002	0.951	0.353
	**5 vs. 30**	**−0.009**	**0.002**	**−3.975**	**0.001**
	**15 vs. 30**	**−0.011**	**0.002**	**−5.25**	**<0.001**
Control	5 vs. 15	0.006	0.003	1.839	0.081
	**5 vs. 30**	**−0.009**	**0.003**	**−2.87**	**0.014**
	**15 vs. 30**	**−0.015**	**0.003**	**−4.983**	**<0.001**
Predator	5 vs. 15	−0.002	0.003	−0.478	0.638
	**5 vs. 30**	**−0.009**	**0.003**	**−2.753**	**0.036**
	**15 vs. 30**	**−0.007**	**0.003**	**−2.442**	**0.04**
Mass growth rate					
Averaged	**5 vs. 15**	**0.002**	**0**	**3.937**	**0.001**
	**5 vs. 30**	**−0.001**	**0**	**−2.653**	**0.015**
	**15 vs. 30**	**−0.003**	**0**	**−6.96**	**<0.001**
Control	**5 vs. 15**	**0.002**	**0.001**	**2.777**	**0.012**
	**5 vs. 30**	**−0.002**	**0.001**	**−3.125**	**0.008**
	**15 vs. 30**	**−0.004**	**0.001**	**−6.194**	**<0.001**
Predator	**5 vs. 15**	**0.002**	**0.001**	**2.79**	**0.017**
	5 vs. 30	0.001	0.001	−0.643	0.528
	**15 vs. 30**	**−0.002**	**0.001**	**−3.649**	**0.007**
Length at metamorphosis					
Averaged	5 vs. 15	−0.021	0.034	−0.606	0.546
	**5 vs. 30**	**−0.234**	**0.032**	**−7.32**	**<0.001**
	**15 vs. 30**	**−0.213**	**0.02**	**−10.417**	**<0.001**
Control	5 vs. 15	0.046	0.049	0.943	0.348
	**5 vs. 30**	**−0.2**	**0.046**	**−4.381**	**<0.001**
	**15 vs. 30**	**−0.246**	**0.029**	**−8.521**	**<0.001**
Predator	5 vs. 15	−0.088	0.048	−1.835	0.069
	**5 vs. 30**	**−0.267**	**0.045**	**−5.991**	**<0.001**
	**15 vs. 30**	**−0.18**	**0.029**	**−6.214**	**<0.001**
Mass at metamorphosis					
Averaged	5 vs. 15	0.003	0.01	0.33	0.743
	**5 vs. 30**	**−0.061**	**0.009**	**−6.407**	**<0.001**
	**15 vs. 30**	**−0.064**	**0.007**	**−9.145**	**<0.001**
Control	5 vs. 15	0.009	0.014	0.602	0.55
	**5 vs. 30**	**−0.053**	**0.013**	**−3.91**	**<0.001**
	**15 vs. 30**	**−0.061**	**0.01**	**−6.199**	**<0.001**
Predator	5 vs. 15	−0.002	0.014	−0.141	0.888
	**5 vs. 30**	**−0.069**	**0.013**	**−5.159**	**<0.001**
	**15 vs. 30**	**−0.067**	**0.01**	**−6.734**	**<0.001**
Time to metamorphosis					
Averaged	5 vs. 15	−0.065	0.032	−2.036	0.125
	5 vs. 30	−0.042	0.03	−1.416	0.235
	15 vs. 30	0.023	0.019	1.179	0.239
Control	5 vs. 15	−0.052	0.046	−1.117	0.396
	5 vs. 30	−0.019	0.043	−0.446	0.656
	15 vs. 30	0.033	0.028	1.174	0.396
Predator	5 vs. 15	−0.078	0.044	−1.783	0.168
	5 vs. 30	−0.065	0.041	−1.589	0.168
	15 vs. 30	0.013	0.027	0.487	0.626
Survival to metamorphosis					
Averaged	5 vs. 15	0.069	0.515	0.133	0.894
	5 vs. 30	0.495	0.495	0.999	0.477
	15 vs. 30	0.426	0.343	1.241	0.477
Control	5 vs. 15	0	0.723	0	1
	5 vs. 30	0.778	0.716	1.087	0.416
	15 vs. 30	0.778	0.501	1.553	0.361
Predator	5 vs. 15	0.137	0.732	0.187	0.875
	5 vs. 30	0.211	0.684	0.309	0.875
	15 vs. 30	0.074	0.469	0.158	0.875

**Table 3:** Comparisons between groups with absent and present predators regarding the growth rate and metamorphic traits of Chinhai spiny newt larvae within each density treatment. Significant differences (*p* < 0.05) are highlighted in bold.

Density Treatments	Estimate	SE	t/z Value	*p* Value
Length growth rate				
5	0.001	0.003	0.322	0.75
**15**	**0.008**	**0.003**	**2.823**	**0.012**
30	0.001	0.003	0.282	0.782
Mass growth rate				
**5**	**0.002**	**0.001**	**2.429**	**0.023**
**15**	**0.002**	**0.001**	**2.644**	**0.018**
30	0	0.001	0.099	0.922
Length at metamorphosis				
5	−0.095	0.059	−1.606	0.11
15	0.038	0.034	1.135	0.265
30	−0.028	0.023	−1.207	0.264
Mass at metamorphosis				
5	−0.018	0.017	−1.092	0.278
15	−0.008	0.011	−0.715	0.483
30	−0.002	0.009	−0.27	0.793
Time to metamorphosis				
5	−0.071	0.055	−1.3	0.194
15	−0.045	0.032	−1.41	0.159
30	−0.026	0.022	−1.186	0.236
Survival to metamorphosis				
5	0	0.886	0	1
15	−0.137	0.524	−0.262	0.794
30	0.566	0.443	1.278	0.201

**Table 4:** Means and standard deviations for the growth and metamorphic traits of larval Chinhai spiny newts were conducted between the density treatments and predator treatments.

Predator Treatments	Density Treatments	Length Growth Rate (cm/Day)	Mass Growth Rate (g/Day)	Length at Metamorphosis (cm)	Mass at Metamorphosis (g)	Time to Metamorphosis (Day)	Metamorphosis/Total
Control	5	0.053 ± 0.01	0.011 ± 0.002	3.397 ± 0.193	0.350 ± 0.043	39 ± 2	N = 16/20 (80%)
	15	0.059 ± 0.006	0.013 ± 0.002	3.443 ± 0.141	0.358 ± 0.041	37 ± 2	N = 51/60 (88%)
	30	0.044 ± 0.005	0.009 ± 0.001	3.197 ± 0.179	0.297 ± 0.045	38 ± 3	N = 111/120 (92.5%)
Predator	5	0.051 ± 0.007	0.010 ± 0.002	3.492 ± 0.2	0.367 ± 0.055	42 ± 4	N = 17/20 (85%)
	15	0.050 ± 0.008	0.012 ± 0.001	3.405 ± 0.157	0.366 ± 0.044	39 ± 2	N = 52/60 (86.67%)
	30	0.043 ± 0.005	0.009 ± 0.001	3.225 ± 0.173	0.299 ± 0.045	39 ± 3	N = 105/120 (87.5%)

## Data Availability

The datasets generated and analyzed during the current study are available on Science DB at https://doi.org/10.57760/sciencedb.18364.

## Supplementary Materials

The following supporting information can be downloaded at: https://www.mdpi.com/article/10.3390/ani14101510/s1, Table S1: Pairwise comparisons were conducted to assess the effect of density treatments on the initial body length and body mass of Chinhai spiny newt larvae within each predator treatment group; Table S2: Comparisons between groups with absent and present predators regarding the initial body length and body mass of Chinhai spiny newt larvae within each density treatment; Table S3: The effect of density treatments, predator treatments, and their interaction on the length and mass of Chinhai spiny newt larvae on the 21st day, presented as type III analysis-of-deviance tables. Significant differences (*p* < 0.05) between different groups are highlighted in bold; Table S4: Pairwise comparisons were conducted to evaluate the impact of density treatments on body length and body mass of Chinhai spiny newt larvae on the 21st day within each predator treatment group; Table S5: Comparisons between groups with absent and present predators regarding the body length and body mass on the 21st day of Chinhai spiny newt larvae within each density treatment.
