# Supplementary material for: Density Mediates the Predator-Induced Growth and Metamorphic Plasticity of Chinhai Spiny Newt Larvae
Source: Animals (Basel). 2024 May 20;14(10):1510. doi: 10.3390/ani14101510 (PMC11117270; doi:10.3390/ani14101510)
Supplement: Supplementary file 1 [file animals-14-01510-s001.zip › animals-3003933-supplementary.pdf]

Supplementary Material

Table S1. Pairwise comparisons were conducted to assess the effect of density treatments on the initial body length and body mass of Chinhai spiny newt larvae within each predator treatment group.

**Predator treatments**	**Contrast**	**Estimate**	**SE**	***t* Value**	***p* Value**
Initial length					
Averaged	low vs medium	0.045	0.025	1.811	0.255
	low vs high	0.016	0.025	0.645	0.527
	medium vs high	-0.029	0.023	-1.24	0.35
Control	low vs medium	0.025	0.035	0.721	0.719
	low vs high	-0.002	0.035	-0.049	0.962
	medium vs high	-0.027	0.033	-0.818	0.719
Predator	low vs medium	0.064	0.035	1.84	0.242
	low vs high	0.034	0.035	0.96	0.364
	medium vs high	-0.031	0.033	-0.935	0.364
Initial mass					
Averaged	low vs medium	0.006	0.003	2.214	0.059
	low vs high	0.006	0.003	2.427	0.059
	medium vs high	0.001	0.002	0.222	0.827
Control	low vs medium	0.005	0.004	1.523	0.216
	low vs high	0.007	0.004	1.877	0.216
	medium vs high	0.001	0.003	0.369	0.717
Predator	low vs medium	0.006	0.004	1.608	0.204
	low vs high	0.006	0.004	1.555	0.204
	medium vs high	0	0.003	-0.055	0.957

Table S2. Comparisons between groups with absent and present predators regarding the initial body length and body mass of Chinhai spiny newt larvae within each density treatment.

**Density treatments**	**Estimate**	**SE**	***t* Value**	***p* Value**
Initial length				
low	0.022	0.037	0.59	0.561
medium	-0.017	0.033	-0.528	0.605
high	-0.014	0.033	-0.411	0.687
Initial mass				
low	-0.001	0.004	-0.365	0.719
medium	-0.002	0.003	-0.484	0.635
high	0	0.003	-0.06	0.953

Table S3. The effect of density treatments, predator treatments, and their interaction on the length and mass of Chinhai spiny newt larvae on the 21th day, presented as type III analysis-of-deviance tables. Significant difference ($p < 0.05$) between different groups are highlighted in bold.

**Response**	**Fixed effect**	X^2	***df***	***P***
Length on the 21th day	**Density treatments**	**40.998**	**2**	**<0.001**
	**Predator treatments**	**4.024**	**1**	**0.045**
	Density treatments * Predator treatments	3.755	2	0.153
Mass on the 21th day	**Density treatments**	**44.158**	**2**	**<0.001**
	Predator treatments	**7.129**	**1**	**0.008**
	Density treatments * Predator treatments	3.516	2	0.172

Table S4. Pairwise comparisons were conducted to evaluate the impact of density treatments on body length and body mass of Chinhai spiny newt larvae on the 21th day within each predator treatment group.

**Predator treatments**	**Contrast**	**Estimate**	**SE**	***t* Value**	***p* Value**
Length growth					
Averaged	low vs medium	0.088	0.044	2.021	0.057
	**low vs high**	**-0.169**	**0.044**	**-3.859**	**0.001**
	**medium vs high**	**-0.257**	**0.041**	**-6.32**	**<0.001**
Control	**low vs medium**	**0.146**	**0.061**	**2.374**	**0.028**
	**low vs high**	**-0.188**	**0.061**	**-3.057**	**0.009**
	**medium vs high**	**-0.334**	**0.058**	**-5.793**	**<0.001**
Predator	low vs medium	0.031	0.062	0.499	0.623
	**low vs high**	**-0.15**	**0.062**	**-2.405**	**0.039**
	**medium vs high**	**-0.181**	**0.058**	**-3.144**	**0.019**
Mass growth					
Averaged	**low vs medium**	**0.046**	**0.011**	**4.236**	**0.001**
	low vs high	-0.021	0.011	-1.973	0.063
	**medium vs high**	**-0.067**	**0.01**	**-6.522**	**<0.001**
Control	**low vs medium**	**0.046**	**0.015**	**2.986**	**0.011**
	**low vs high**	**-0.039**	**0.015**	**-2.535**	**0.02**
	**medium vs high**	**-0.084**	**0.015**	**-5.764**	**<0.001**
Predator	**low vs medium**	**0.046**	**0.015**	**3.005**	**0.011**
	low vs high	-0.004	0.015	-0.269	0.791
	**medium vs high**	**-0.051**	**0.015**	**-3.459**	**0.01**

Table S5. Comparisons between groups with absent and present predators regarding the body length and body mass on the 21th day of Chinhai spiny newt larvae within each density treatment.

Density treatments	Estimate	SE	*t* Value	*p* Value
Length on the 21th day				
low	0.042	0.066	0.632	0.533
**medium**	**0.156**	**0.058**	**2.715**	**0.016**
high	0.004	0.058	0.066	0.948
Mass on the 21th day				
**low**	**0.036**	**0.016**	**2.223**	**0.036**
**medium**	**0.035**	**0.015**	**2.385**	**0.03**
high	0.001	0.015	0.08	0.937
